# Cerebral Microbleeds Assessment and Quantification in COVID-19 Patients With Neurological Manifestations

**DOI:** 10.3389/fneur.2022.884449

**Published:** 2022-05-23

**Authors:** Angela Napolitano, Alberto Arrigoni, Anna Caroli, Mariangela Cava, Andrea Remuzzi, Luca Giovanni Longhi, Antonino Barletta, Rosalia Zangari, Ferdinando Luca Lorini, Maria Sessa, Simonetta Gerevini

**Affiliations:** ^1^Department of Neuroradiology, ASST Papa Giovanni XXIII, Bergamo, Italy; ^2^Bioengineering Department, Istituto di Ricerche Farmacologiche Mario Negri IRCCS, Bergamo, Italy; ^3^Radiology Unit, S. Giacomo Hospital, Novi Ligure, Italy; ^4^Department of Management, Information and Production Engineering, University of Bergamo, Bergamo, Italy; ^5^Neurosurgical Intensive Care Unit, Department of Anesthesia and Critical Care Medicine, ASST Papa Giovanni XXIII, Bergamo, Italy; ^6^Research Foundation, ASST Papa Giovanni XXIII, Bergamo, Italy; ^7^Department of Emergency and Critical Care Area, ASST Papa Giovanni XXIII, Bergamo, Italy; ^8^Department of Neurology, ASST Papa Giovanni XXIII, Bergamo, Italy

**Keywords:** susceptibility-weighted imaging (SWI), neuro-COVID, inflammation, MRI, cerebral microbleeds (CMBs)

## Abstract

It is increasingly acknowledged that Coronavirus Disease 2019 (COVID-19) can have neurological manifestations, and cerebral microbleeds (CMBs) have been observed in this setting. The aim of this study was to characterize CMBs patterns on susceptibility-weighted imaging (SWI) in hospitalized patients with COVID-19 with neurological manifestations. CMBs volume was quantified and correlated with clinical and laboratory parameters. The study included patients who were hospitalized due to COVID-19, exhibited neurological manifestations, and underwent a brain MRI between March and May 2020. Neurological, clinical, and biochemical variables were reported. The MRI was acquired using a 3T scanner, with a standardized protocol including SWI. Patients were divided based on radiological evidence of CMBs or their absence. The CMBs burden was also assessed with a semi-automatic SWI processing procedure specifically developed for the purpose of this study. Odds ratios (OR) for CMBs were calculated using age, sex, clinical, and laboratory data by logistic regression analysis. Of the 1,760 patients with COVID-19 admitted to the ASST Papa Giovanni XXIII Hospital between 1 March and 31 May 2020, 116 exhibited neurological symptoms requiring neuroimaging evaluation. Of these, 63 patients underwent brain MRI and were therefore included in the study. A total of 14 patients had radiological evidence of CMBs (CMBs+ group). CMBs+ patients had a higher prevalence of CSF inflammation (*p* = 0.020), a higher white blood cell count (*p* = 0.020), and lower lymphocytes (*p* = 0.010); the D-dimer (*p* = 0.026), LDH (*p* = 0.004), procalcitonin (*p* = 0.002), and CRP concentration (*p* < 0.001) were higher than in the CMBs- group. In multivariable logistic regression analysis, CRP (OR = 1.16, *p* = 0.011) indicated an association with CMBs. Estimated CMBs volume was higher in females than in males and decreased with age (Rho = −0.38; *p* = 0.18); it was positively associated with CRP (Rho = 0.36; *p* = 0.22), and negatively associated with lymphocytes (Rho = −0.52; *p* = 0.07). CMBs are a frequent imaging finding in hospitalized patients with COVID-19 with neurological manifestations and seem to be related to pro-inflammatory status.

## Introduction

In December 2019, the new severe acute respiratory syndrome coronavirus 2 (SARS-CoV-2) caused an outbreak of severe pneumonia and coronavirus-related diseases (COVID-19) in China, which rapidly spread globally. Italy and the Lombardy region, in particular, were severely affected (1).

Although the predominant symptoms are respiratory, associated neurological manifestations—such as stroke, headache, altered mental status, epileptic seizures, movement disorders, and hyposmia/ageusia—have increasingly been acknowledged, with a rising number of studies detecting central nervous system abnormalities in patients affected by COVID-19 (2–4).

Concurrently, microhemorrhages (cerebral microbleeds, CMBs) have been observed radiologically in the brain. Whether these reported alterations are coincidental occurrences, non-specific implications of a systemic disorder, common complications of a severe infectious disease, or a direct consequence of the viral infection, remains open. It is fully acknowledged that SARS-CoV-2 can enter and damage endothelial cells in the lungs, heart, and kidneys by binding angiotensin-converting enzyme 2 (ACE2) and activating inflammatory and thrombotic pathways. A similar cascade could be involved in cerebral damage observed in COVID-19 patients (5–7). In particular, slow blood flow in cerebral microvessels allows the viral spike protein to interact with ACE2 receptors in capillaries in the endothelium. A damaged endothelial lining would favor viral access to the brain, where the virus can damage neuronal cells expressing ACE2, even in the absence of substantial inflammation. The endothelial ruptures in cerebral capillaries may lead to cerebral hemorrhage (5). CMBs appear on susceptibility-weighted imaging (SWI) as parenchymal punctate hypointensities, potentially unrelated to ischemia and macro hemorrhage (8) and this can be observed in a variety of conditions, especially in patients with acute respiratory distress syndrome (9) or widespread intravascular coagulation (10). CMBs can be observed in the subcortical white matter (WM) and splenium of the corpus callosum (CC) in critically ill patients who have experienced prolonged respiratory failure and periods of hypoxemia (11–13).

In this context, the aim of the study was to characterize CMBs patterns on SWI and investigate possible associations between the incidence of CMBs and potential risk factors, neurological symptoms and clinical and laboratory data from patients hospitalized due to COVID-19 with neurological manifestations. In addition, CMBs volume was quantified and correlated with clinical and laboratory parameters.

## Materials and Methods

### Study Design and Patient Selection

Patients who were hospitalized due to COVID-19, who exhibited neurological manifestations and underwent a brain MRI between March and May 2020 were eligible for inclusion. Patients included in the study were divided into groups based on radiological evidence of CMBs (CMBs+ group) or the absence of CMBs (CMBs-). The local ethics committee approved the collection and scientific use of the patients' data as part of a larger observational study protocol (reg 2020-144). Informed consent was obtained from patients or provided by their next of kin or legal guardians.

### COVID-19 Diagnosis

The COVID-19 diagnosis was confirmed using an algorithm based on local guidelines that included: (1) real-time reverse-transcriptase polymerase-chain-reaction (RT-PCR) on at least 1 nasopharyngeal swab; or (2) RT-PCR on bronchoalveolar lavage in case of high clinical suspicion of SARS-CoV-2 infection in spite of negative test results from at least two nasopharyngeal swabs performed at least 24 h apart; or (3) in the case of negative RT-PCR for SARS-COV-2, typical clinical presentation during the epidemic phase (fever, dry cough, and dyspnea) with radiological evidence of interstitial pneumonia.

### Clinical and Laboratory Data

Clinical and laboratory data were extracted from patients' electronic medical records in the Hospital Information System. Clinical data included demographic information, past medical history, presenting symptoms and neurological symptoms, and the need for ventilatory support. The laboratory data considered were levels of white blood cells, lymphocytes, hemoglobin (Hb), platelet maximum and minimum counts, C-reactive protein (PCR), procalcitonin, creatinine, lactate dehydrogenase (LDH), prothrombin time (PT), activated partial thromboplastin time (aPTT), D-dimer, fibrinogen and, when available, cerebrospinal fluid (CSF) analysis. Inflammatory CSF was defined as pleocytosis and elevated protein concentration. Only data from laboratory tests performed within 3 days of the brain MRI were considered.

### MRI Acquisition and Visual Assessment

All brain MRI scans were acquired at the ASST Papa Giovanni XXIII hospital in Bergamo, Italy, using a General Electric 3 Tesla MRI scanner (Discovery MR 750w GEM).

The brain MRI acquisition protocol included pre-contrast coronal T2-weighted, pre-contrast axial T1-weighted and post-contrast 3D T1-weighted, pre- and post-contrast sagittal FLAIR, diffusion-weighted and tensor imaging, susceptibility-weighted imaging (SWI) and perfusion imaging. SWI acquisition was performed axially, with the following parameters: TR = 38 ms, TE = 26 ms, matrix size = 512 × 512, slice thickness = 1.3 mm.

All anonymized MRI scans were evaluated independently by two experienced neuroradiologists (SG, MC) while two more junior colleagues who were blinded to the clinical data (AB, AN) reviewed the abnormalities to classify the findings. When their assessments diverged the cases were reviewed by a third person, and decisions were based on consensus in all cases. SWI scans were reviewed to assess and quantify microbleeds. CMBs were described in terms of number and location, and the involvement of specific brain areas, such as the corpus callosum (CC), internal capsules (IC), and cerebellar peduncles was considered. CC microbleeds were further classified as involving all of the segments or only the splenium. The shape of CMBs could be defined as dot-like or linear, the latter resembling a vascular structure. Superficial siderosis was also noted. In the presence of CMBs on SWI, the FLAIR and DWI sequences were analyzed to detect white matter abnormalities or restricted diffusion lesions. Leukoencephalopathy was defined as diffuse confluent white matter FLAIR hyperintensities, more than expected for age-related microangiopathy on the basis of visual qualitative assessment.

### MRI Processing and CMBs Quantification

**Figure 1:**
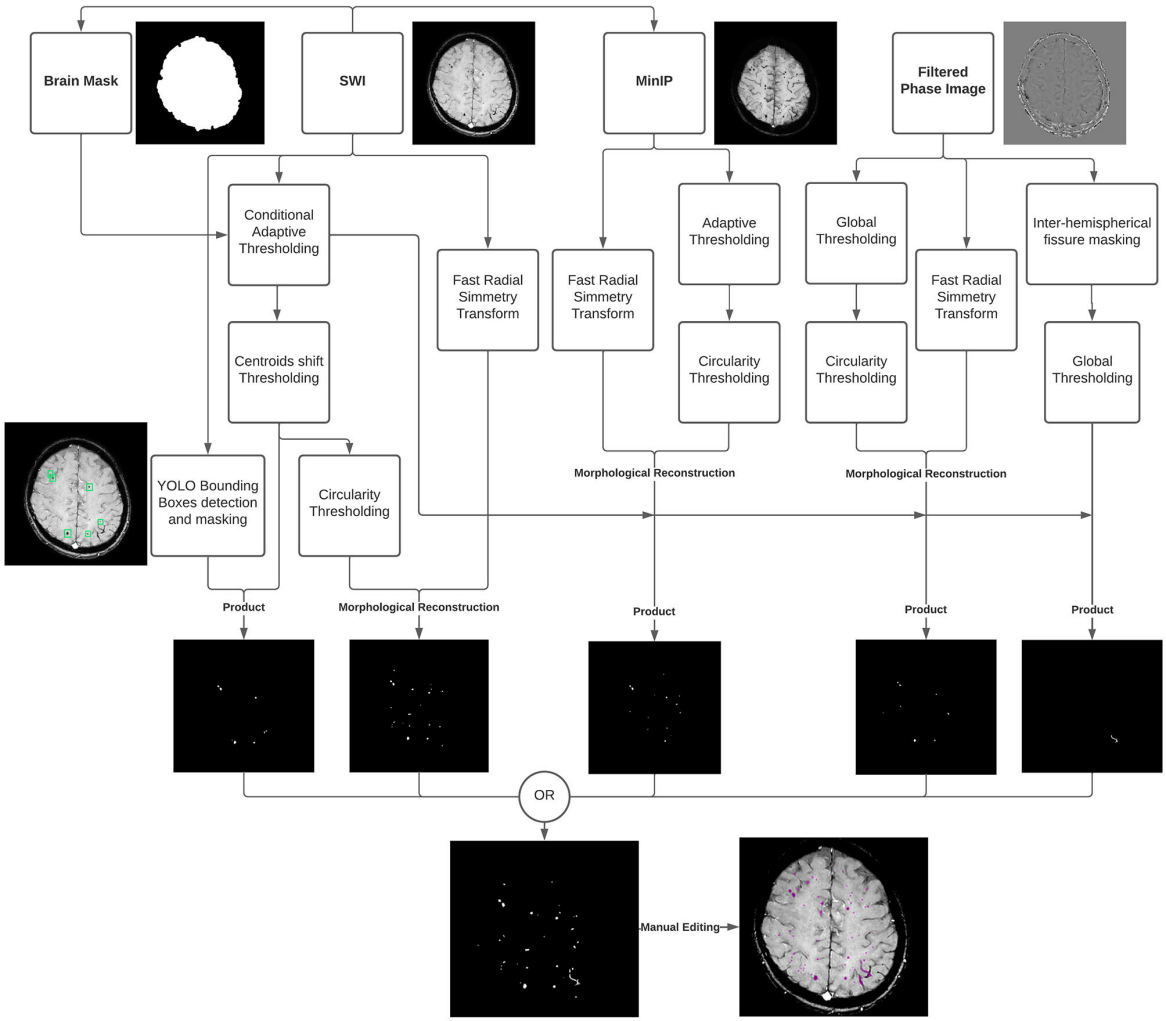
Diagram summarizing the cerebral microbleeds (CMBs) segmentation algorithm developed and used in the study. The algorithm uses the susceptibility-weighted imaging (SWI) sequence along with the Filtered Phase sequence and a brain mask, as input, followed by a Minimum Intensity Projection (MinIP) generated from the SWI scans. A Fast Radial Symmetry Transform (FRST) technique is used to detect regions of interest based on local radial symmetry. Likewise, a deep learning (YOLO) detection algorithm is used to identify CMBs bounding boxes on SWI scans. The resulting binary masks are combined with the output of intensity thresholding and geometric feature extraction techniques to provide possible CMBs segmentations. Different intensity thresholding approaches are used according to the different input images and pathways. Specifically, a global approach is used for the flat gray Filtered Phase images; adaptive thresholding is used on the MinIP images to deal with the presence of different brightness regions picking the locally darkest particles, associated with the lesions; and the Conditional Adaptive Thresholding method is applied to the SWI scans to compute local thresholds inside the previously defined brain mask. A centroid criterion is additionally applied to consecutive slices to avoid the segmentation of the vessels' orthogonal sections resembling dot-shaped and spheric CMBs. An optional step based on global thresholding and inter-hemispherical fissure masking of the Filtered Phase stack makes it possible to segment tubular-shaped CMBs in addition to the round lesions. The resulting segmentations results are finally combined via the OR operator, and the outcome can be manually refined to fix possible segmentation inaccuracies.

The CMBs burden was assessed on SWI scans using an in-house semi-automatic processing procedure (Figure 1), using: ImageJ/Fiji, version 1.53c (https://imagej.nih.gov/ij), Python, version 3.7 (http://www.python.org) and MATLAB, version R2019a (Natick, MA, USA).

A brain mask was first created using SWI scans with an intensity-based region-growing approach, and the Minimum Intensity Projection (MinIP) was generated to differentiate between CMBs and circular vessel sections.

Secondly, a pre-trained YOLO deep learning model was used to detect and mask CMBs bounding boxes. Taking CMBs morphology into account and based on previous findings (14), the actual segmentation procedure relied on the Fast Radial Symmetry Transform (FRST) technique (15).

FRST was combined with an intensity-based adaptive thresholding approach, so-called “Conditional Adaptive Thresholding” (16), which made it possible to compute pixel-specific thresholds inside the brain mask. An optional step based on thresholding of the Filtered Phase stack made it possible to identify and also include linear-shaped lesions.

The resulting segmentation could be manually edited using 3D Slicer software to fix possible inaccuracies in concordance with a neuroradiologist (AN) and CMBs quantification was finally performed by multiplying the total segmented area by the space between slices and the number of slices.

### Statistical Analysis

Comparisons between CMBs+ and CMBs- patients were performed using the Mann–Whitney or Fisher tests as appropriate. Odds ratios (OR) for CMBs were identified in age, sex, clinical and laboratory data using logistic regression analysis. Univariate analyses were performed first; all variables with nearly significant contributions (*p* < 0.1) at univariate analysis were included in the multivariate analysis, alongside age and sex. The model was subsequently reduced using an AIC stepwise model selection technique. In patients with CMBs, the distribution of CMBs total volume by each binary variable (sex, intensive care, CSF findings, comorbidities, and clinical symptoms) was displayed using boxplots. The correlation between CMBs total volume and age, time to MRI, duration of hospitalization or invasive mechanical ventilation and each clinical and laboratory variable was assessed using Spearman correlation. Statistical significance was set at *p* < 0.05. All statistical analyses were performed using R software (R Core Team, Vienna, Austria), version 4.0.5.

## Results

**Figure 2:**
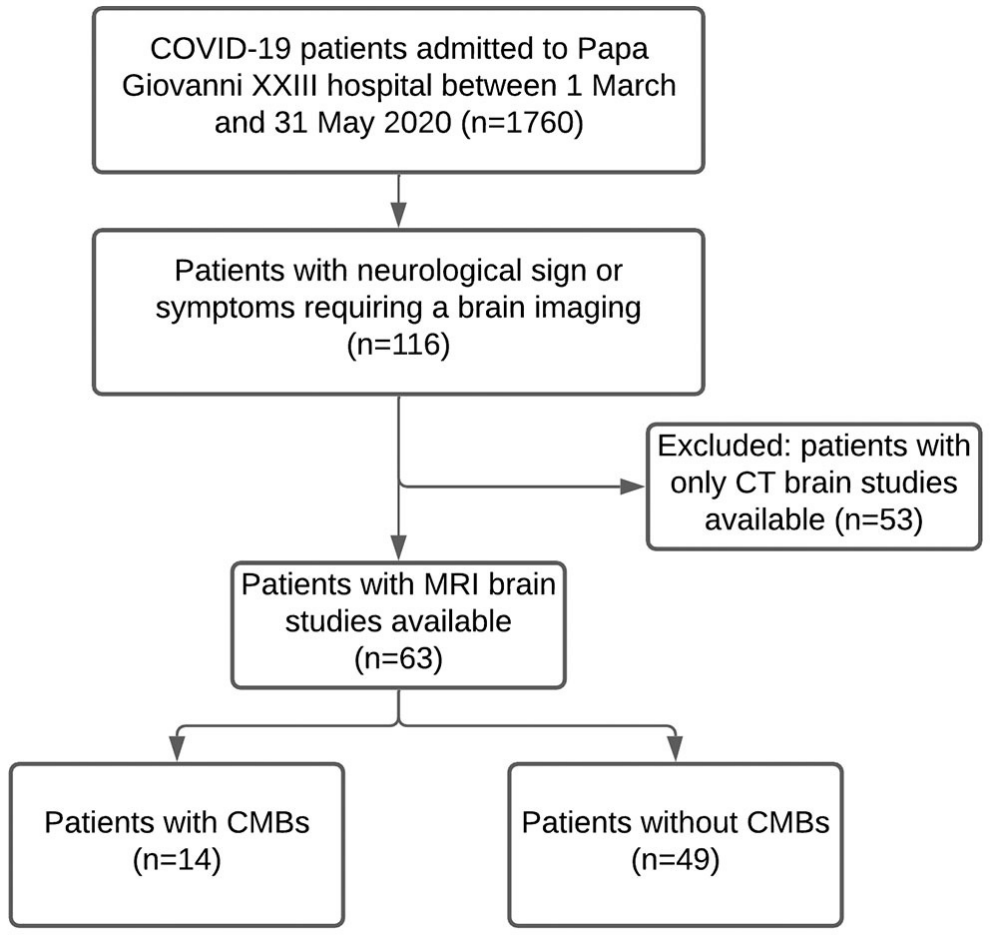
Flow chart of the study participants.

Out of 1,760 patients hospitalized due to COVID-19, 116 exhibited neurological symptoms and required brain imaging. Fifty-three of them underwent a brain CT scan, while the remaining 63 underwent a brain MRI scan and were therefore eligible for the study. Of these patients, 14 had radiological evidence of CMBs (CMBs+ group), while 49 did not (CMBs- group). Figure 2 is a flow chart that describes the study participants.

### CMBs Subgroups Characterization and Risk Factors

Socio-demographic features and clinical and laboratory data for the two subgroups are summarized in Table 1.

**Table 1:** Demographic and clinical characteristics of 63 patients hospitalized due to COVID-19 and exhibiting neurological symptoms.

		**Total** **(*N* = 63)**	**CMBs+** **(*n* = 14)**	**CMBs-** **(*n* = 49)**	***p-*value**
Sex (M)		39 (62%)	10 (71%)	29 (59%)	0.538
Age (years)		64 (56–73)	62 (56–73)	64 (56–72)	0.741
**Comorbidities**					
Dyslipidemia		4 (6%)	1 (7%)	3 (6%)	1.000
Heart disease		18 (29%)	5 (36%)	13 (27%)	0.517
Diabetes		12 (19%)	0 (0%)	12 (24%)	0.053
Hypertension		27 (43%)	7 (50%)	20 (41%)	0.557
COPD		5 (8%)	1 (7%)	4 (8%)	1.000
Cancer		4 (6%)	2 (14%)	2 (4%)	0.211
Past CVA or TIA		5 (8%)	3 (21%)	2 (4%)	0.068
**Clinical presentation**					
Dyspnea		27 (43%)	8 (57%)	19 (39%)	0.239
Head trauma		4 (6%)	1 (7%)	3 (6%)	1.000
Cough		22 (35%)	6 (43%)	16 (33%)	0.534
Fever		34 (54%)	10 (71%)	24 (49%)	0.224
Confusion		24 (38%)	8 (57%)	16 (33%)	0.124
Visual impairment		3 (5%)	0 (0%)	3 (6%)	1.000
Headache		8 (13%)	0 (0%)	8 (16%)	0.182
Stroke		9 (14%)	1 (7%)	8 (16%)	0.669
Ataxia		3 (5%)	0 (0%)	3 (6%)	1.000
Seizure		10 (16%)	2 (14%)	8 (16%)	1.000
Anosmia or ageusia		2 (3%)	1 (7%)	1 (2%)	0.398
Neuropathy		5 (8%)	1 (7%)	4 (8%)	1.000
Focal deficit		18 (29%)	1(7%)	17 (35%)	0.051
Coma		7 (11%)	2 (14%)	5 (10%)	0.646
**Laboratory tests**					
Inflammatory CSF		9 (14%)	5 (36%)	4 (8%)	**0.020**
White blood cells (10^3^/ μl)	no. with data	60	13	47	
	Median [IQR]	11.1 (8.3–15.8)	14.7 (12.7–19.4)	10.4 (7.8–14.8)	**0.020**
Lymphocytes (10^3^/μl)	no. with data	59	13	46	
	Median [IQR]	0.7 (0.5–1.1)	0.5 (0.3–0.7)	0.8 (0.6–1.3)	**0.010**
Hemoglobin (g/dl)	no. with data	60	13	47	
	Median [IQR]	10.6 (8.1–13.1)	8.1 (7.7–10.0)	11.4 (8.8–13.5)	**0.013**
Platelet (min) (10^3^/μl)	no. with data	58	13	45	
	Median [IQR]	174 (124–239)	136 (110–174)	188 (144–244)	0.086
Platelet (max) (10^3^/μl)	no. with data	60	13	47	
	Median [IQR]	332 (278–451)	329 (300–427)	334 (274–452)	0.907
C-reactive protein (mg/dL)	no. with data	59	13	46	
	Median [IQR]	12.0 (3.7–26.6)	27.1 (18.3–36.5)	7.8 (3.2–17.9)	**<0.001**
Procalcitonin (ng/ml)	no. with data	32	8	24	
	Median [IQR]	0.85 (0.19–1.33)	1.7 (1.28–12.20)	0.5 (0.10–1.04)	**0.002**
Creatinine (mg/dL)	no. with data	55	13	42	
	Median [IQR]	1.0 (0.8–1.4)	1.2 (0.8–1.8)	0.9 (0.8–1.2)	0.146
LDH (IU/l)	no. with data	56	13	43	
	Median [IQR]	404 (250–601)	583 (468–715)	384 (234–540)	**0.004**
PT (s)	no. with data	56	13	43	
	Median [IQR]	1.1 (1.0–1.3)	1.1 (1.0–1.3)	1.1 (1.0–1.3)	0.806
aPTT (s)	no. with data	58	13	45	
	Median [IQR]	1.1 (1.0–1.3)	1.1 (1.0–1.3)	1.0 (1.0–1.3)	*0.540*
D-dimer (ng/ml)	no. with data	52	12	40	
	Median [IQR]	1,590 (578–4,345)	5,135 (1,023–8,910)	1,211 (520–3,565)	**0.026**
Fibrinogen (mg/dL)	no. with data	43	11	32	
	Median [IQR]	730 (485–960)	704 (496–932)	762 (489–952)	0.900
Invasive mechanical ventilation		21 (33%)	9 (64%)	12 (24%)	**0.009**
/ duration		16 (9–26)	19 (12–26)	15 (9–24)	0.749
Chest X-ray COVID-19 positivity		29/40 (73%)	9/14(64%)	20/26 (77%)	0.469
Chest CT COVID-19 positivity		41/55 (75%)	9/13 (69%)	32/42 (76%)	0.719
Pulmonary embolism		3/18 (17%)	1/5 (20%)	2/13 (15%)	1.000
Hospitalizations (days)	no. with data	60	14	46	
	Median [IQR]	23 (11–42)	41 (24–54)	20 (9–35)	**0.028**
Time to MRI (days)	no. with data	61	14	47	
	Median [IQR]	7 (3–22)	31 (14–55)	6 (3–13)	**0.002**
FAZEKAS				
	−0	39 (62%)	6 (43%)	33 (67%)	0.205
	−1	16 (25%)	6 (43%)	10 (20%)	
	−2	6 (10%)	2 (14%)	4 (8%)	
	−3	2 (3%)	0 (0%)	2 (4%)	
Leukoencephalopathy		3 (5%)	3 (21%)	0 (0%)	**0.009**
Exitus (Death)		4/61 (7%)	1/14 (7%)	3/47 (6%)	1

*Patients were divided into groups based on the presence or absence of cerebral microbleeds on MRI scans (CMBs+ and CMBs-, respectively).*

*Data are reported as median (IQR; continuous/numerical variables) or number (%; binary/categorical variables). p-values are computed using the Mann-Whitney test (continuous variables) or Fisher's exact test (binary or categorical variables).*

*aPTT, activated partial thromboplastin time; COPD, Chronic obstructive pulmonary disease; CRP, C-reactive protein; CSF, cerebrospinal fluid; CT, computed tomography; CVA, cerebrovascular accident; LDH, lactate dehydrogenase; MRI, magnetic resonance imaging; PT, prothrombin time; TIA, transient ischemic attack.*

*Bold means statistically significant*.

Both CMBs+ and CMBs- patients were predominantly male, and there were no significant differences between the two groups regarding age. Leukoencephalopathy was reported in three CMBs+ and in none of the patients with no CMBs (21 vs. 0%; *p* = 0.009). CMBs+ were hospitalized for longer than—patients with no CMBs- (median of 41 vs. 20 days; *p* = 0.028) and also required more frequent invasive mechanical ventilation (64 vs. 24%; *p* = 0.009). CMBs+ underwent MRI significantly later than CMBs- patients (31 vs. 6 days after admission; *p* = 0.002). The two groups also exhibited significant differences in laboratory parameters: CMBs+ patients had a higher prevalence of CSF inflammation (36 vs. 8%; *p* = 0.020), higher white blood cell concentration (14.7 vs. 10.4 10^3^/μl; *p* = 0.020), and a lower lymphocyte concentration than CMBs- patients (0.5 vs. 0.8 10^3^/μl; *p* = 0.010).

In the CMBs+ group, hemoglobin was significantly lower (8.1 vs. 11.4 10^3^/μl; *p* = 0.013), while the D-dimer (5,135 vs. 1,211; *p* = 0.026), LDH (583 vs. 384; *p* = 0.004), procalcitonin (1.7 vs. 0.5; *p* = 0.002), and CRP concentration (27.1 vs. 7.8; *p* < 0.001) were significantly higher than in the CMBs- group. On the other hand, the two groups were not significantly different in terms of symptoms at presentation and comorbidities (Table 1).

Invasive mechanical ventilation, duration of hospitalization, the time between admission and MRI acquisition, inflammatory CSF, lymphocytes, Hb, CRP, and LDH were found to be significant OR for the radiological evidence of CMBs in the univariate logistic regression analysis (Table 2). Among those parameters, the main risk factors included in the reduced multivariate model were age, sex, past CVA or TIA, focal deficit, positive CSF, LDH, and CRP. The only OR that remained significant in multivariate analysis was CRP concentration [OR = 1.16 (95% CI, 1.05–1.34), *p* = 0.011] (Table 2).

**Table 2:** Demographic, clinical, and laboratory risk factors for cerebral microbleeds MRI finding in 63 patients hospitalized due to COVID-19 and exhibiting neurological symptoms.

	**Univariable**		**Multivariable**	
	**OR (95% CI)**	***p*-value**	**OR (95% CI)**	***p*-value**
Sex (M)	1.72 (0.50–7.00)	*0.409*	7.01 (0.76–139)	0.1261
Age (per year)	1.00 (0.93–1.05)	*0.807*	1.09 (0.96–1.30)	*0.260*
**Comorbidities**				
Dyslipidemia	1.18 (0.06–10.10)	*0.890*		
Heart disease	1.54 (0.41–5.36)	*0.504*		
Diabetes	-			
Hypertension	1.45 (0.43–4.87)	*0.541*		
COPD	0.86 (0.04–6.51)	*0.901*		
Cancer	3.92 (0.43–35.50)	*0.194*		
Past CVA or TIA	6.41 (0.96–53.30)	*0.056*	52.2 (1.63–7,158)	*0.050*
**Clinical presentation**				
Dyspnea	2.11 (0.64–7.32)	*0.226*		
Head trauma	1.18 (0.06–10.10)	*0.890*		
Cough	1.55 (0.44–5.22)	*0.482*		
Fever	2.60 (0.76–10.50)	*0.145*		
Confusion	2.75 (0.82–9.68)	*0.103*		
Visual impairment	-			
Headache	-			
Stroke	0.39 (0.02–2.45)	*0.401*		
Ataxia	-			
Seizure	0.85 (0.19–4.00)	*0.854*		
Anosmia or ageusia	3.69 (0.14– 97.70)	*0.367*		
Neuropathy	0.87 (0.04–6.51)	*0.901*		
Focal deficit	0.145 (0.01–0.82)	*0.074*	0.13 (0.01–1.43)	*0.144*
Coma	1.47 (0.19–7.80)	*0.670*		
**Laboratory tests**				
Inflammatory CSF	6.25 (1.40–30.00)	***0.016***	26.7 (0.97–2,547)	*0.081*
White blood cells (10^3^/μl)	1.06 (0.99–1.13)	*0.080*		
Lymphocytes (10^3^/μl)	0.05 (0.00– 0.39)	***0.017***		
Hemoglobin (g/dl)	0.70 (0.50–0.93)	***0.020***		
Platelet (min) (10^3^/ μl)	0.99 (0.98–1.00)	*0.145*		
Platelet (max) (10^3^/ μl)	0.99 (0.99–1.00)	*0.712*		
C-reactive protein (mg/dl)	1.11 (1.05–1.19)	***0.001***	1.16 (1.05–1.34)	***0.011***
Procalcitonin (ng/ml)	1.03 (1.00–1.10)	*0.245*		
Creatinine (mg/dl)	1.31 (0.90–2.05)	*0.160*		
LDH (IU/l)	1.00 (1.00–1.01)	***0.018***	1.00 (1.00–1.01)	*0.200*
PT (s)	1.04 (0.33–2.48)	*0.936*		
aPTT (s)	1.10 (0.51–1.95)	*0.751*		
D-dimer (ng/ml)	1.00 (0.99–1.00)	*0.389*		
Fibrinogen (mg/dl)	0.99 (0.99–1.00)	*0.466*		
Invasive mechanical ventilation	5.55 (1.61– 21.30)	***0.008***		
/ duration	1.00 (0.95–1.05)	*0.941*		
Chest X-ray COVID-19 positivity	0.54 (0.13–2.30)	*0.396*		
Chest CT COVID-19 positivity	0.70 (0.18–3.03)	*0.616*		
Pulmonary embolism	1.38 (0.05–18.9)	*0.814*		
Hospitalizations (days)	1.03 (1.00–1.05)	***0.037***		
Time to MRI (days)	1.04 (1.01–1.06)	***0.005***		
FAZEKAS				
−1	3.30 (0.86–12.9)	*0.080*		
−2	2.75 (0.33–17.9)	*0.299*		
Exitus (Death)	1.13 (0.05–9.69)	0.920		

*Odds ratios, 95% CI, and p-values were first computed for each variable using univariate logistic regression models (left). All variables with significant contributions in univariate analysis were included in the multivariate analysis alongside age and gender, and main risk factors (right) were finally identified by reducing the multivariate model using an AIC stepwise model selection technique. The number of missing data for each variable included in the univariate analysis is reported in Table 1. Only patients with no missing data (n = 56) were included in the multivariate analysis.*

*aPTT, activated partial thromboplastin time; COPD, Chronic obstructive pulmonary disease; CRP, C-reactive protein; CSF, cerebrospinal fluid; CT, computed tomography; CVA, cerebrovascular accident; LDH, lactate dehydrogenase; MRI, magnetic resonance imaging; PT, prothrombin time; TIA, transient ischemic attack.*

*Bold means statistically significant*.

### CMBs Qualitative Assessment

**Figure 3:**
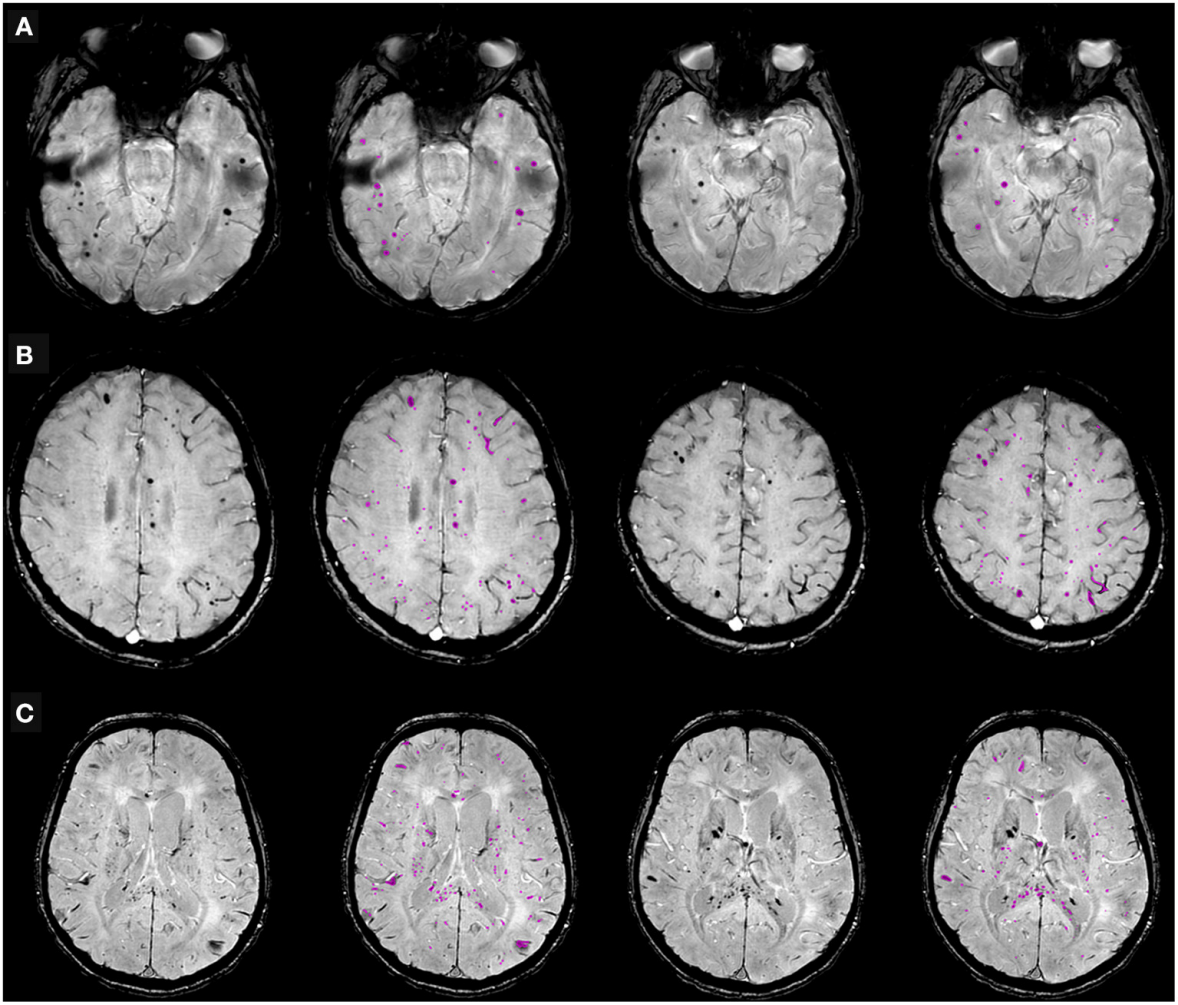
Cerebral microbleeds (CMBs) segmentation on susceptibility-weighted imaging (SWI) in representative patients hospitalized due to COVID-19, with neurological symptoms. **(A)** 68-year old man with typical dot-shaped and spherical CMBs; **(B)** 48-year old woman with the less common ovoid and tubular-shaped lesions in addition to the conventional appearance; **(C)** 55-year old man with CMBs also located in the corpus callosum. Two SWI slices per patient are shown, along with pertinent CMBs segmentation results.

The distribution of CMBs is shown in Table 3 and some examples are shown in Figure 3. CMBs presented as punctuated in all patients; in four patients linear hypointensities were also detected in association with dot-like hypointensities. CMBs mainly involved the juxtacortical white matter and corpus callosum, particularly the splenium. Supratentorial and infratentorial involvement, both in the subcortical and deep WM, was labeled as diffuse. CMBs were observed in all the segments of the CC in 9 (64%) patients, whereas two patients had CMBs involving only the splenium. The infratentorial location was involved in 7/14 patients (50%) with lesions seen in the pons and cerebellar peduncles. The gray matter was mostly spared.

**Table 3:** Distribution of CMBs and leukoencephalopathy.

	**N. of Patients (%)**
**Distribution of leukoencephalopathy**, ***n*** **=** **3**	
Diffuse	3 (100%)
**Distribution of cerebral microbleeds**, ***n*** **=** **14**	
Diffuse	8 (57%)
Lobar	11 (79%)
Pons/cerebellum	7 (50%)
Corpus callosum including splenium	9 (64%)
Splenium only	2 (14%)
Subcortical white matter	12 (86%)
Deep white matter	7 (50%)

CMBs were associated with leukoencephalopathy in three patients, without evidence of WM cytotoxic edema on the DWI sequence; in three cases, small foci of restricted diffusion indicating small ischemic lesions in the “border zone” areas were detectable; in eight cases, microbleeds were the sole imaging pathological finding. No major bleeding was found in CMBs+ patients. All patients with CMBs presented a negative CT concomitant evaluation, no focal or extensive hyperdense lesions were found.

### CMBs Quantitative Assessment

The procedure designed in-house identified and quantified CMBs total volume on the SWI scans (Figure 3).

**Figure 4:**
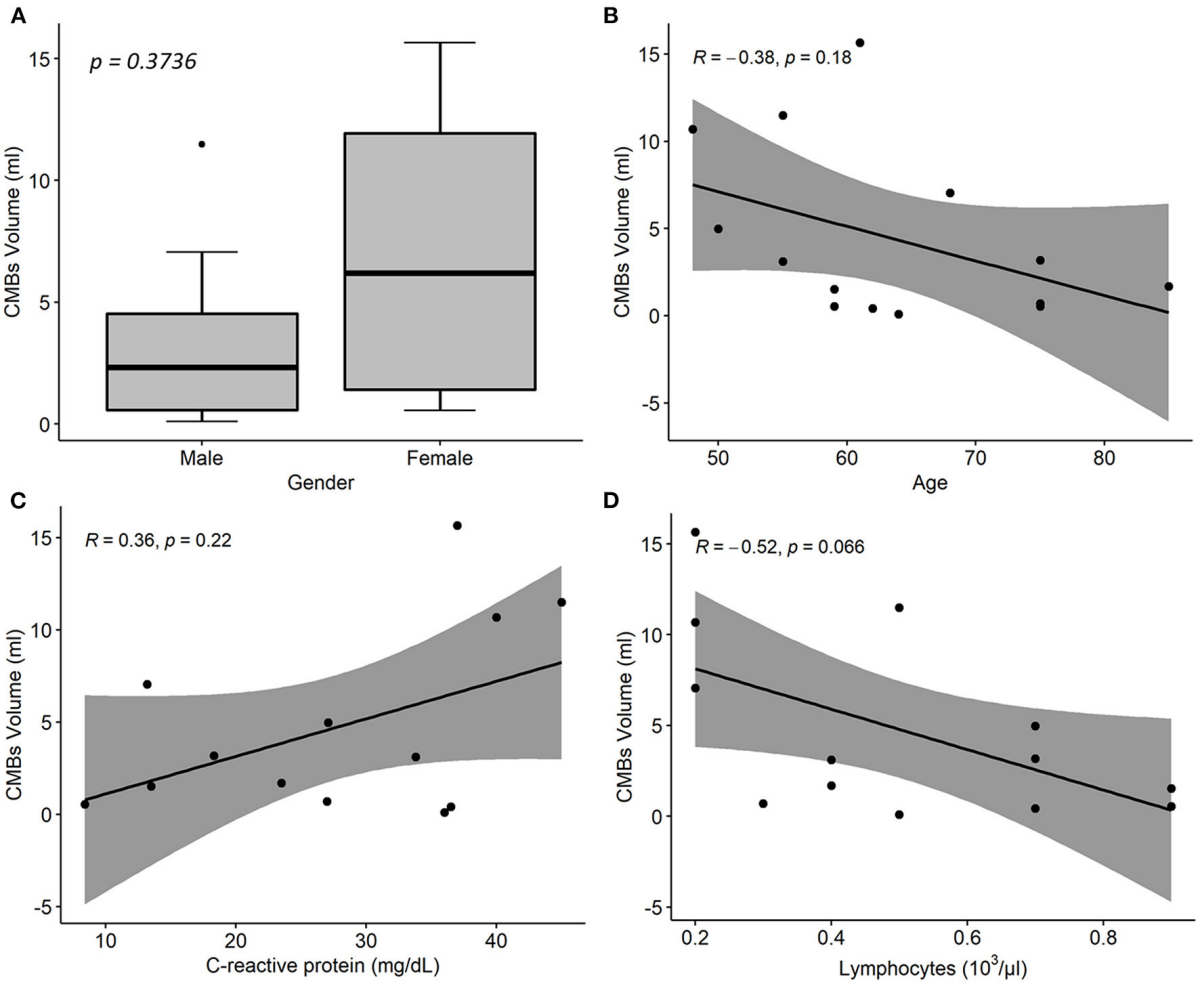
Associations between cerebral microbleeds (CMBs) total volume and descriptive and laboratory parameters in 14 COVID-19 patients with neurological disorders and microhemorrhages detected on susceptibility-weighted images (SWI). **(A)** Distribution of CMBs total volume on SWI by patient sex. *p*-value was assessed by Wilcoxon test. **(B–D)** Linear regression of CMBs total volume on age **(B)**, CRP **(C)**, and lymphocytes concentration values **(C)**. *R* denotes the Spearman correlation coefficient with the pertinent *p*-value. CMBs, cerebral microbleeds; CRP, C-reactive protein.

Estimated CMBs volume was higher in females than in males and decreased with age (Rho = −0.38; *p* = 0.18); it was positively associated with CRP (Rho = 0.36; *p* = 0.22), and negatively associated with lymphocytes (Rho = −0.52; *p* = 0.07) and fibrinogen concentration (Rho = −0.68; *p* = 0.025; Figure 4).

## Discussion

Cerebral microbleeds are an emerging and singular imaging finding in patients with COVID-19 (17–19). CMBs may be secondary to endothelial dysfunction, causing the focal extravasation of red blood cells into the brain. The pathophysiological basis of this in COVID-19 remains a matter of debate, and several hypotheses have been proposed. In the setting of severe COVID-19 infection, hypoxemia-induced brain changes have been postulated as the cause of both leukoencephalopathy and CMBs (20).

We observed a specific pattern of distribution of CMBs in COVID-19, characterized by prominent callosal and juxtacortical involvement, as reported in the literature. This pattern, only detected by blood-sensitive MRI sequences, has been described extensively in critical illness associated-CMBs, a rare condition reported in patients with acute respiratory failure that requires mechanical ventilation, and sometimes ECMO (21, 22). Severe hypoxemia is common in such conditions and could account for CMBs formation but may not be the only cause. In our study, the CMBs-positive group had a higher rate of invasive mechanical ventilation (IMV) compared to patients without this imaging finding. As described by other groups (20, 23, 24), patients with CMBs had critical COVID-19 (i.e., need for IMV in the ICU setting and longer hospitalization).

It is well-documented that SARS-CoV-2 can cause damage to endothelial cells in several organs, such as the lungs, heart, and kidneys, activating inflammatory and thrombotic pathways (6). Endothelial cell infection or monocyte activation, the upregulation of tissue factors, and the release of microparticles, which activate the thrombotic pathway, might occur in SARS-CoV-2, like in other viruses (25, 26).

This phenomenon may explain the high number of acute coronary syndromes or acute myocardial dysfunction without clear culprits, such as lesions observed through angiography, which were considered to be related to myocarditis (27) or acute extensive pulmonary failure with no evidence of acute pulmonary emboli that had dilated peripheral vessels, with 100% of the patients exhibiting perfusion defects in a dual-energy CT (28).

In our study the presence of CMBs was associated with a CSF inflammatory profile; reduced concentrations of Hb and lymphocytes; higher levels of white blood cells, procalcitonin, and LDH and CRP, which correlated positively with CMBS total volume. In the adjusted regression multivariable analysis, the association with CRP levels is confirmed, thus suggesting that inflammation plays a predominant role. The RECOVERY trial has also provided solid evidence of the importance of inflammation in body due to COVID-19 (29).

It is probable that the inflammatory response plays the most important role in inducing damage to the blood-brain barrier (BBB). In a large autopsy cohort study performed in the Netherlands (30), like in other smaller pathology studies, the authors report increased blood-brain barrier (BBB) permeability and rupture and blood red cell extravasation.

The burden of CMBs, as well as their presence, may be important. In our study, the estimated volume of CMBs seems to correlate with the inflammatory status and severity of the disease, as represented by lymphopenia (31). It remains unknown whether this pattern of brain involvement has a similar pathophysiology as neurological symptoms of the long-COVID syndrome.

The limitations of this retrospective single-center study include the limited number of COVID-19 patients with a brain MRI, especially those with radiological evidence of CMBs, and the design of the study, so that caution is required in interpreting the results. Because of the state of the epidemic (32) many steps were limited by the astonishing number of patients treated for COVID-19 pneumonia. Only a fraction of patients were hospitalized, and it is likely that some neurological manifestations were underrecognized in these patients. Lastly, gold standard procedures like brain autopsy were not available.

Furthermore, to our knowledge, this is one of the largest single-center cohorts of consecutive patients with COVID-19 with neurological manifestations. The study period corresponds to the first wave in Italy, one of the first in the world, so no previous exposure to the virus or vaccination could reduce the effect of the disease. MRI studies were performed with a standardized acquisition protocol, which made comparisons more reliable. Finally, this customized semi-automatic segmentation procedure made it possible to quantify CMBs total volume. The implemented solution combined state-of-the-art methods previously used to detect CMBs on SWI scans (14, 33, 34) with the deep learning detection approach (35–37), and an optimized thresholding method, with the techniques supporting each other and providing accurate CMBs segmentation and volume quantification. Since CMBs have been reported in a variety of clinical conditions (38), our CMBs segmentation technique could also be useful in other settings, apart from COVID-19 (e.g., amyloid angiopathy).

In conclusion, patients with COVID-19 may develop a wide range of neurological symptoms, which can be associated with severe and fatal complications. Neuroimaging and MRI, in particular, can reveal brain changes in such patients. CMBs are a frequent imaging finding in critical patients with COVID and seem to be related to pro-inflammatory status. Future larger longitudinal studies are needed to confirm the current findings.

## Data Availability Statement

The raw data supporting the conclusions of this article will be made available by the authors, upon reasonable request.

## Ethics Statement

The studies involving human participants were reviewed and approved by Local Ethics Committee as part of a Larger Observational Study (protocol reg 2020-144). The patients/participants provided their written informed consent to participate in this study.

## Author Contributions

AN and SG: study design. AN, AA, AC, and LL: analysis and interpretation of data. AN, AA, and AC: drafting of the manuscript. AN, AA, AC, LL, MC, AR, AB, RZ, FL, MS, and SG: critical revision of the manuscript. All authors contributed in the approval of the final version for submission.

## Funding

The authors declare that this study received funding from Brembo S.p.a. (Curno, Bergamo, Italy), under the main funding project Progetto TrexUno. The funder was not involved in the study design, collection, analysis, interpretation of data, the writing of this article or the decision to submit it for publication.

## Conflict of Interest

The authors declare that the research was conducted in the absence of any commercial or financial relationships that could be construed as a potential conflict of interest.

## Publisher's Note

All claims expressed in this article are solely those of the authors and do not necessarily represent those of their affiliated organizations, or those of the publisher, the editors and the reviewers. Any product that may be evaluated in this article, or claim that may be made by its manufacturer, is not guaranteed or endorsed by the publisher.
